# Biomaterial Material Applications in Postoperative Surgical Fields of Uvulopalatopharyngoplasty: A Comparative Study

**DOI:** 10.3390/jfb14070337

**Published:** 2023-06-26

**Authors:** Tsung-Che Yu, Ting-Chieh Huang, Po-Yueh Chen, Chun-Che Shih, Wei-Wen Chang

**Affiliations:** 1 Department of Otolaryngology, Wan Fang Hospital, Taipei Medical University, Taipei 116, Taiwan; 108244@w.tmu.edu.tw (T.-C.Y.); 107156@w.tmu.edu.tw (T.-C.H.); 110120@w.tmu.edu.tw (P.-Y.C.); 2 Division of Cardiovascular Surgery, Department of Surgery, Wan Fang Hospital, Taipei Medical University, Taipei 116, Taiwan; ccshih@w.tmu.edu.tw; 3 Division of General Surgery, Department of Surgery, Wan Fang Hospital, Taipei Medical University, Taipei 116, Taiwan

**Keywords:** Uvulopalatopharyngoplasty, tissue glue, platelet-rich plasma, polyglycolic acid, pain management, hemostasis

## Abstract

This retrospective study compared the effectiveness of different materials used in Uvulopalatopharyngoplasty (UPPP) for snoring or obstructive sleep apnea treatment, focusing on the impact on bleeding control, pain control, and healing ability. The study population comprised 213 patients who underwent UPPP at Wan-Fang Hospital between July 2018 and October 2022 divided into four groups based on the postoperative material used: No Material Use Group, Tissue Glue Group, Platelet-Rich Plasma (PRP) Group, and Polyglycolic Acid (PGA) Sheet Group. Results showed significant differences in operation time and intraoperative bleeding amount among the groups, with the Tissue Glue Group demonstrating the shortest operation time. While no significant differences in postoperative pain at 24 h were observed, PRP and PGA Sheet groups exhibited lower average pain scores in cases with higher pain levels. Postoperative complications and emergency room visits due to pain or bleeding varied among the groups, with the No Material Use Group having the highest incidence, although no statistical significance was achieved. This study provides insights into the potential benefits of using advanced materials in UPPP, guiding future research and clinical practice to improve patient care and outcomes.

## 1. Introduction

Uvulopalatopharyngoplasty (UPPP) is a surgical procedure widely used to address obstructive sleep apnea, characterized by the resection of excess tissue in the throat to increase the airway size and reduce the air resistance that leads to sleep apnea [1,2,3]. Despite its effectiveness, the operation is frequently associated with various postoperative complications, such as pain, bleeding, and slow healing, posing a significant challenge to the medical field [4,5,6,7]. Therefore, using advanced materials as dressings plays an important role in airway surgical field control.

The development and utilization of functional materials in medical procedures offer a promising avenue for improving surgical outcomes. In the postoperative surgical field, various materials have been extensively evaluated to optimize patient outcomes. For instance, a multitude of barrier materials, including 67 unique adhesion barrier agents, are being explored to prevent postoperative surgical adhesions. These materials, encompassing both natural and synthetic types, have shown promising results in animal studies, with some further validated in human trials [8]. In the field of otorhinolaryngology, several biomaterials have been developed to enhance the outcomes of endoscopic sinus surgery. These materials, which include chitosan, gelatin, hyaluronic acid, and starch-derived materials, are designed to prevent postoperative bleeding, facilitate wound healing, and reduce inflammation. They have demonstrated potential efficacy in nasal packing, improving wound healing, and patient-reported outcomes [9]. The biomaterials can even be fabricated into self-expanding sinus stents used in functional endoscopic sinus surgery [10]. Materials can also be utilized in the application of 3D bioprinting technologies in reconstruction and tissue engineering for head and neck surgery [11]. These materials, engineered with specific properties tailored to the application, could potentially enhance the effectiveness of UPPP. They may reduce postoperative bleeding by promoting clotting, alleviate pain through targeted drug release, or expedite healing by facilitating tissue regeneration. It has been proposed that local hemostasis maneuvers, including the placement of Surgicel or topical thrombin, can be used during these airway operations [12]. Similarly, applying fibrin glue to the operative sites in tonsillectomy and adenoidectomy can provide effective hemostasis and seal with good systemic and local compatibility [13]. The application of hemostasis bandages has also been shown to enhance the hemostasis results in recovery times better than previously reported for this common operation in patients receiving adenotonsillectomy [14]. Besides hemostasis, applying materials postoperatively to the surgical field was also more effective in pain control than traditional management [15].

The choice of materials used during the surgery plays a crucial role in managing these complications, making it a critical factor worthy of a thorough investigation. However, the comparative effectiveness of these different materials remains uncertain, particularly concerning bleeding control, pain control, and healing ability. This uncertainty hinders the optimization of the surgical procedure and the subsequent postoperative recovery, necessitating a rigorous and systematic analysis of the available options.

The present study aims to compare the effectiveness of different materials used in UPPP, focusing specifically on those employed at Wan-Fang Hospital from July 2018 to October 2022. Through this comparative analysis, we seek to elucidate the impacts of these materials on bleeding control, pain control, and healing ability. By identifying the most effective materials and shedding light on their respective advantages and disadvantages, we can guide future research and clinical practice, ultimately enhancing the quality of patient care and improving the lives of those affected by obstructive sleep apnea.

## 2. Materials and Methods

### 2.1. Study Design and Data Collection

This study was a retrospective analysis of patient data obtained from the Department of Otolaryngology at Wan-Fang Hospital between July 2018 and October 2022. Ethical clearance was procured from TMUJIRB (approval number: N202305085), and all data were anonymized to maintain patient confidentiality.

### 2.2. Participants and Baseline Data

The study population consisted of patients who had undergone Uvulopalatopharyngoplasty (UPPP) within the study period. The inclusion criteria were adult patients (>18 years) diagnosed with snoring or obstructive sleep apnea and treated with UPPP. Exclusion criteria were patients with incomplete medical records, those who had simultaneous surgeries that could confound the study outcomes, and those with a history of previous throat surgery. Baseline data collected included age, gender, Body Mass Index (BMI), hemoglobin level, platelet level, prothrombin time (PT), and activated partial thromboplastin time (aPTT).

### 2.3. Materials Used in the Surgical Field

All materials used in the study are clinically approved and used as received without any modifications.

#### 2.3.1. Fibrin Tissue Glue

Tissel^®^ is used as tissue glue in this study and is a fibrin sealant with a strong clinical safety and efficacy record, used as an adjunct to hemostasis. It is particularly useful in surgical sites where traditional methods like sutures and cautery are impractical. Effective across a wide range of surgical specialties, Tissel^®^ helps control bleeding in heparinized patients and those on antiplatelet drugs and creates hemostasis on poor tissue characteristics like friable tissue. It should not be used for severe or brisk arterial bleeding [16].

#### 2.3.2. Polyglycolic Acid Sheet

The Neoveil^®^ sheet is a bioabsorbable polyglycolic acid (PGA) felt, often used in surgeries where sutures or staple lines provide inadequate fixation. It is designed to offer additional strength, support tissue regeneration and healing, and potentially reduce the risk of complications. The sheet gradually degrades through hydrolysis before being fully absorbed and metabolized by the body [17].

#### 2.3.3. PRP

Aeon Biotherapeutics Corp’s Acti-PRP^®^ is used in this study. The process involves drawing blood or bone marrow from a patient and centrifuging it to create a concentrated liquid of key elements. This concentrated liquid is then applied to the treatment area to stimulate healing. Notably, the product is designed such that no additional drugs or artifacts are added; only the patient’s own blood is used [18]. The biological mechanisms that underpin the applications of PRP in wound healing include the combined action of platelet-derived growth factors and cytokines, along with the role of plasma-derived fibrillar, antioxidant, and homeostatic factors. Regenerative treatments involving PRP consist of personalized and non-standardized methods, meaning the quality of PRP can vary depending on several endogenous factors (e.g., age, gender, concomitant medication, disease-associated systemic factors, and nutrition) and exogenous factors (such as anticoagulants and cellular composition) [19].

### 2.4. Grouping of Patients

Patients were categorized into four groups based on the postoperative material used during their UPPP procedure: “No Material Use Group (Abbreviated as NONE in figures and table)”, “Tissue Glue Group”, “PRP Group”, and “PGA Sheet Group”.

### 2.5. Data Parameters

The parameters compared across the groups were operation time, bleeding amount, postoperative pain, and postoperative emergency room visits due to pain or bleeding.

Postoperative pain was evaluated using a 10-point visual analog scale (VAS), with scores recorded at 24 h postoperatively. Operation time was recorded from the start to the end of the surgical procedure. The bleeding amount was quantified through the intraoperative suction container and surgical sponges. Postoperative emergency room visits due to pain or bleeding are regarded as complication indicators and were documented through medical records and compared.

### 2.6. Statistical Analysis

Data were analyzed using SPSS version 26.0. Descriptive statistics were generated for all study variables. Continuous variables were compared using ANOVA, and categorical variables were compared using Fisher’s exact test as required. A *p*-value less than 0.05 was considered statistically significant.

## 3. Results

The study sample comprised a total of 213 patients who underwent UPPP at Wan-Fang Hospital between July 2018 and October 2022. These patients were divided into four groups: No Material Use Group (*n* = 132), Tissue Glue Group (*n* = 46), PRP Group (*n* = 33), and PGA Sheet Group (*n* = 2).

### 3.1. Baseline Demographics

Baseline demographics and clinical characteristics, including age, gender, BMI, hemoglobin level, platelet level, PT, and aPTT, were comparable across all groups. There were no statistically significant differences between the groups in these parameters (*p* > 0.05), indicating a balanced distribution across the different groups (Table 1).

### 3.2. Operation Time

**Figure 1:**
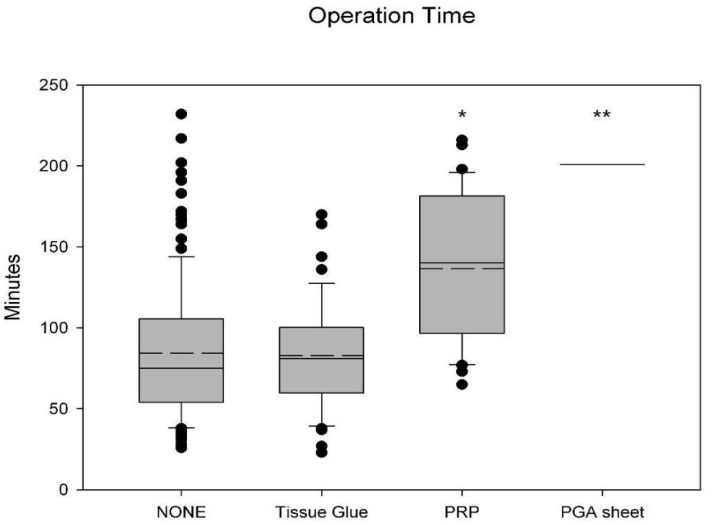
Comparison of operation times among different treatment groups. The No Material Use group had an operation time of 84.4 ± 42.2 min. The operation time for the Tissue Glue group (*n* = 46) was the shortest, averaging 82.8 ± 33.6 min, with no significant difference compared to the No Material Use group (*n* = 132) (*p* = 0.81). The operation times for the PRP group (*n* = 33) and PGA (*n* = 2) sheet group were significantly longer, averaging 136.5 ± 44.2 min and 201 min, respectively, both with a significant difference compared to the No Material Use group (*, ** *p* < 0.001, mean value shown as long dashed line, ⚫: data points outside Minimum and Maximum).

As shown in Figure 1, there were significant differences in the operation time between the No Material Use group and the tissue glue group (*p* = 0.81). The operation time of the PRP group is significantly higher than the No Material Use Group (136.5 ± 44.2 versus 84.4 ± 42.2 min, *p* < 0.001). The operation time of the PGA sheet group is also significantly higher than the No Material Use Group (201 versus 84.4 ± 42.2 min, *p* < 0.001). The Tissue Glue Group, on average, had the shortest operation time (82.8 ± 33.6 min).

### 3.3. Bleeding Amount

**Figure 2:**
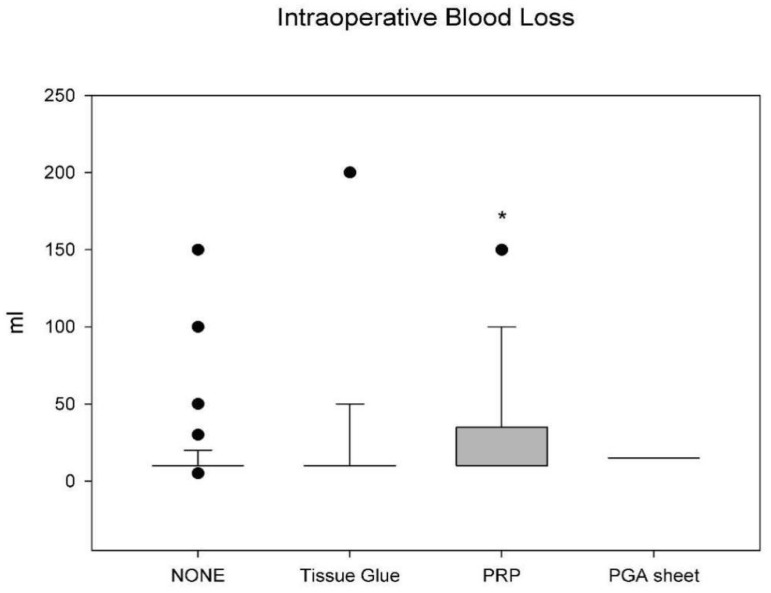
Comparison of average intraoperative bleeding volumes across the different groups. The graph shows the mean and standard deviation of the bleeding volumes for the No Material Use group (*n* = 132) (14.13 ± 16.59 mL), the Tissue Glue group (*n* = 46) (19.78 ± 30.37 mL), the PGA Sheet group (*n* = 2) (15.0 ± 7.07 mL), and the PRP group (*n* = 33) (27.88 ± 35.33 mL). The PRP group’s significantly higher average bleeding volume compared to the No Material Use group is marked with * (*p* = 0.036) (⚫: data points outside Minimum and Maximum).

Variations were observed in the intraoperative bleeding volumes across the groups, yet no statistically discernible differences were found between the No Material Use group, the Tissue Glue group, and the PGA Sheet group (Figure 2). The PRP group exhibited the highest average volume of bleeding (27.88 ± 35.33 mL), which was statistically significant when compared to the No Material Use group (14.13 ± 16.59 mL) with a *p*-value of 0.036. This was followed by the Tissue Glue group (19.78 ± 30.37 mL) and the PGA Sheet group (15.0 ± 7.07 mL).

### 3.4. Postoperative Pain

**Figure 3:**
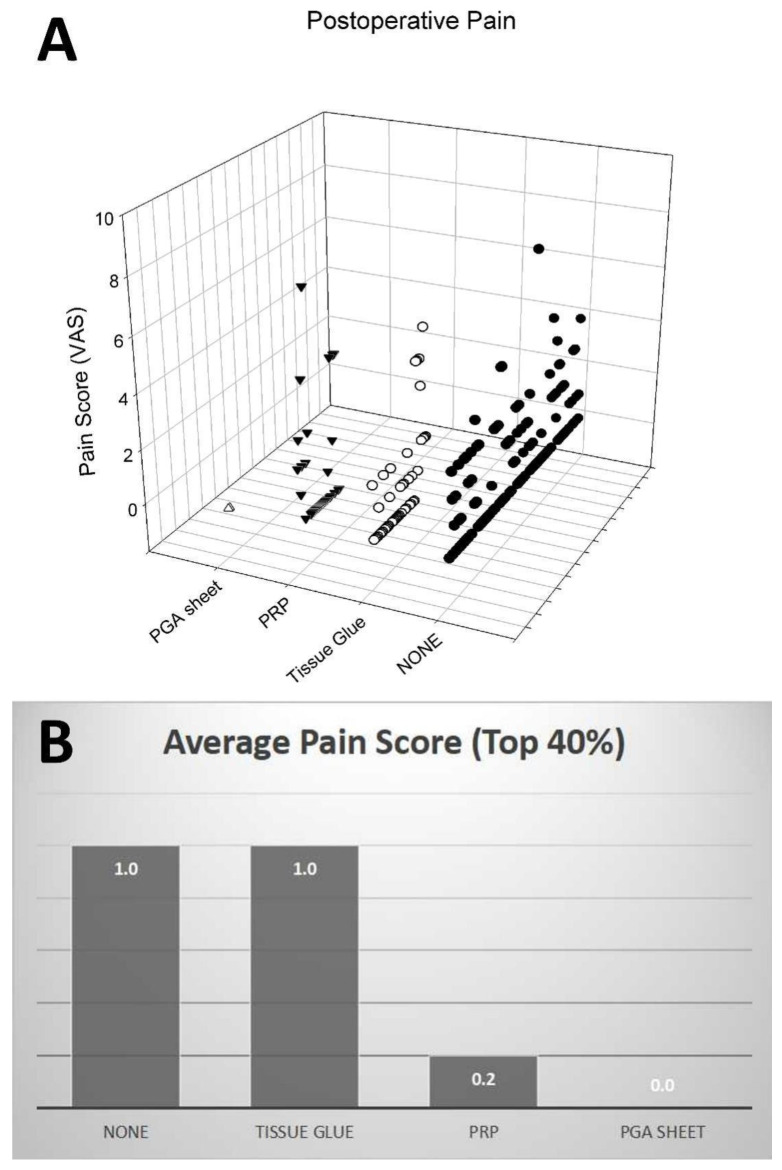
(**A**): Scattered plot illustrating the distribution of Visual Analog Scale (VAS) pain scores at 24 h’ post-operation across four groups: No Material Use, Tissue Glue, Platelet-Rich Plasma (PRP), and Polyglycolic Acid (PGA) Sheet. No statistically significant differences were observed between the groups due to low case numbers. Each symbol in each group represents one single individual. (**B**): Bar chart representing the distribution of VAS pain scores at 24 h’ post-operation for the top 40% of cases with higher pain scores. In these cases, PRP and PGA Sheet groups showed lower average pain scores (0.2 and 0.0, respectively), suggesting the potential benefits of these materials in managing postoperative pain in more severe cases.

The assessment of postoperative pain at 24 h, as gauged by the Visual Analog Scale (VAS), revealed no significant differences among the various treatment groups due to the limited number of cases (Figure 3A). The No Material Use Group had a VAS score of 1.08 ± 1.47, while the Tissue Glue Group reported a score of 1.06 ± 1.60. The Platelet-Rich Plasma (PRP) Group recorded a VAS score of 1.33 ± 2.10. These variations were not statistically significant despite the noted differences in the average VAS scores (*p* > 0.05). Interestingly, the Polyglycolic Acid (PGA) Sheet Group reported no postoperative pain, with a VAS score of 0 ± 0.

Further analysis of the top 40% of cases with higher VAS pain scores at 24 h post-operation revealed an intriguing trend (Figure 3B). The average pain scores for this subset of patients were No Material Use Group at 1.0, Tissue Glue Group at 1.0, Platelet-Rich Plasma (PRP) Group at 0.2, and Polyglycolic Acid (PGA) Sheet Group at 0.0. Notably, the PRP and PGA Sheet groups demonstrated superior performance in these more severe cases with lower recorded pain scores. This suggests a potential benefit of PRP and PGA Sheet in managing postoperative pain in patients experiencing higher levels of discomfort.

### 3.5. Postoperative Complications and Emergency Room Visits

**Figure 4:**
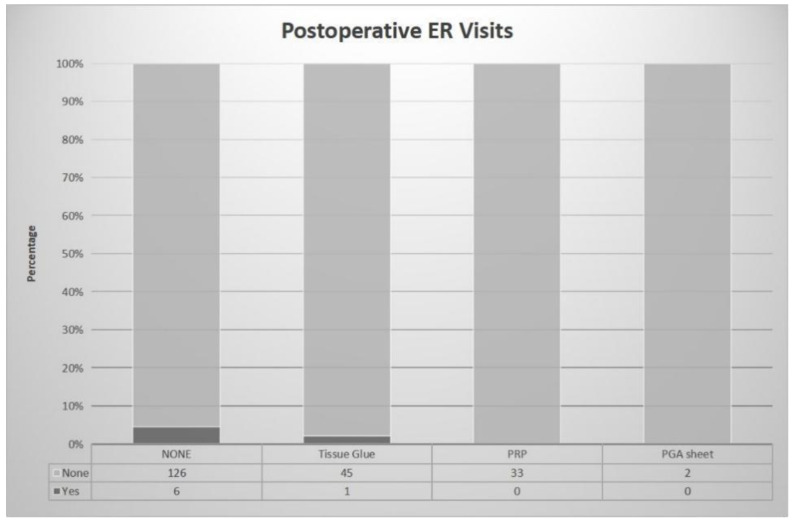
Comparison of postoperative complications leading to emergency room visits among different treatment groups. The No Material Use Group demonstrated the highest rate of emergency room visits due to postoperative pain or bleeding. The Tissue Glue group showed a lower rate, while the PGA Sheet and PRP groups reported no cases. Note that statistical significance was not achieved due to the low number of cases.

Postoperative complications that led to emergency room visits due to pain or bleeding demonstrated variability among the groups, although no statistical significance was achieved due to low case numbers (Figure 4). The No Material Use group had the highest number of cases visiting the emergency room, with 6 out of 132 patients (approximately 4.5%). This was followed by the Tissue Glue group, where 1 out of 46 patients (approximately 2.2%) required an emergency room visit. The PGA Sheet group and the PRP Group reported no cases of emergency room visits due to postoperative complications. Despite the lack of statistical significance, it is noteworthy that the incidence appears higher in the No Material Use Group.

## 4. Discussion

Our study found that the type of material used in UPPP significantly influenced operation time, intraoperative bleeding, postoperative pain, and complications including emergency room visits due to pain or bleeding. The PRP Group showed the most favorable results in postoperative pain and postoperative complication rate, while the Tissue Glue Group had the shortest operation time and less bleeding. These findings suggest that the choice of material could substantially affect the surgical outcomes in UPPP, and the findings are of paramount importance as they underscore the substantial influence of functional materials on the surgical outcomes of UPPP, a procedure widely performed to alleviate the symptoms of obstructive sleep apnea.

Particularly noteworthy were the findings concerning Tissue Glue and PRP. The Tissue Glue Group demonstrated the shortest operation time with low intraoperative bleeding, suggesting that this material may enhance surgical efficiency and improve bleeding control. On the other hand, the PRP Group exhibited the most favorable outcomes in terms of postoperative pain and complication rates, which implies a potentially superior role of PRP in patient comfort and recovery. However, a relatively higher intraoperative blood loss in the PRP group might be an issue of concern.

The choice of materials used post-UPPP holds significant clinical implications. UPPP is a common surgical intervention for obstructive sleep apnea, a condition that severely impairs the quality of life. Hence, optimizing the outcomes of UPPP is crucial to treat the condition effectively and minimizing postoperative discomfort and complications, thereby enhancing patient recovery and satisfaction.

The use of functional materials in UPPP has the potential to influence various facets of the surgical outcome. From reducing operation time and minimizing bleeding to controlling postoperative pain and reducing complications, the choice of material can significantly impact the overall success of the surgery and the subsequent recovery process. Identifying the most effective materials in these aspects can potentially streamline the surgical process, improve patient outcomes, and increase overall satisfaction.

The review of the existing body of literature on this topic to date has yielded mixed results. Some studies have highlighted the benefits of using specific materials, such as tissue glue in UPPP, citing reduced operative times and bleeding. Fibrin-based glues have been used in various surgical procedures, including tonsillectomy and Uvulopalatopharyngoplasty (UPPP). Fibrin-based biomaterials contain fibrinogen and thrombin, which initiate the formation of fibrin clots. These clots are large mesh-like polymers that trap platelets and red blood cells, promoting wound healing and hemostasis. Products such as Tisseel^®^, Tissucol^®^, and Quixil^®^ have been used in tonsillectomy procedures, although their effectiveness varies. Some studies found that Quixil^®^ could reduce pain and prevent bleeding, while a review of hemostatic glues concluded that fibrin glues were not effective in reducing the severity of pain and bleeding in tonsillectomy. As for UPPP, while tissue glues may have the potential for use, no explicit information is available regarding their routine use in this type of surgery [20]. In our study, the use of the fibrin glue seems to be a very “balanced” performance that resulted in a postoperative pain control and complication control, although slightly inferior to the PRP group, but still significantly better than the none-used group, without any elongation of the operation time. Notably, the Tissue Glue Group presented with the shortest operation time and low intraoperative bleeding, suggesting that this material may contribute to surgical efficiency and better bleeding control.

Others have advocated for using PRP, attributing its advantages to its potential wound-healing properties and pain-reduction capabilities. Platelet-rich plasma (PRP) is a concentrated blood product used in various medical fields to enhance tissue regeneration and wound healing. Recently, its use has been explored in airway surgeries such as tonsillectomy and Uvulopalatopharyngoplasty (UPPP). PRP contains high concentrations of growth factors, which are thought to improve the healing process after surgery. In airway surgeries, PRP may promote better wound healing, reduce postoperative pain, and potentially decrease recovery time [21]. A study regarding the short-term postoperative outcomes of pediatric tonsillectomy patients demonstrated the superiority of tonsillectomy with PRP over tonsillectomy alone in terms of effectiveness in reducing post-tonsillectomy pain and improving appetite status, together with a lower requirement for analgesia and a reduced risk of post-tonsillectomy bleeding during the first ten postoperative days [22]. The benefit is shown in many other trials with similar settings [23]. Regarding postoperative pain in our current study, the PRP Group demonstrated the most favorable results, with the lowest scores on the VAS 24 h after the operation. This may suggest that PRP has potent analgesic effects, which can contribute to patient comfort and recovery postoperatively.

Limited studies have focused on using the polyglycolic acid sheet in the field of airway surgery. Polyglycolic acid (PGA) sheets have been used in oral surgeries such as partial glossectomy for their potential to reduce postoperative complications. It has been shown that covering wounds with autologous fibrin glue and PGA sheets may help avoid the risks of viral infection and allergic reactions in partial glossectomy cases [24,25]. Interestingly, when used in tonsillectomies, a study by Niyaguchi et al. showed a completely different result in their study the postoperative pain measured before breakfast was significantly more severe in the PGA sheet–attached side than the nonattached side, and the authors reported the negative effects of PGA sheeting on post-tonsillectomy pain [26]. However, although very limited cases, our study’s result seems to support the use of the PGA sheet as the effect in controlling postoperative pain and complications seems promising. Future studies with more cases would be needed to reveal the true effectiveness of this protocol.

The longer operation time in the PRP group might be due to the longer preparation time, as blood has to be drawn from the patient and processed through a series of centrifuge protocols. After separating the cellular content, the gel-stage PRP has to be appropriately handled, and all these take a certain of time and thus must be considered if cost-effectiveness is a consideration [27]. This phenomenon is also observed in the PGA sheet group, although with only a few cases, the operation time is significantly more prolonged, which could be because quite several meticulous stitching is required for a proper address of the PGA sheet onto the operation field. In other surgical fields, especially in gastroenterology, unique suture methods or staplers can be used to facilitate the fixation of the PGA sheet [28,29]. Considering the result revealed in this study, further developing similar PGA sheet suturing devices might be necessary for upper airway surgeries to diminish the concern of prolonged operation time.

The significantly higher average intraoperative blood loss observed in the PRP group could be attributed to several factors. One potential explanation is the lack of additional hemostatic components within the PRP itself. Hemostatic agents play a crucial role in minimizing blood loss during surgery by promoting blood clotting and tissue sealing, thereby reducing bleeding. If the PRP used in this study lacked these hemostatic components, it would be less effective in controlling intraoperative bleeding, resulting in a greater blood loss volume than in other groups. Additionally, a prolonged operation time could also contribute to the increased blood loss in the PRP group. If the procedures involving PRP application were more time-consuming than those of the other groups, this could explain the observed increase in intraoperative blood loss. Longer surgeries inherently carry a higher risk of blood loss due to the extended tissue exposure and manipulation period. Nevertheless, a study investigating the risk of hemorrhage following oral surgical operations and testing the usefulness of the autologous platelet gel in order to control hemostasis have encouraged the use of PRP when carrying out surgical treatments on patients who are undergoing coumarin therapy [30]. Further studies are required to definitively establish the causes of this observed difference in intraoperative blood loss among the groups.

The implications of our findings extend to various aspects of clinical practice. Our results offer evidence-based guidance for surgeons in selecting functional materials during UPPP. If the goal is to minimize operative time and bleeding, tissue glue may be the optimal choice. Conversely, PRP could be the preferred material if postoperative pain control and postoperative complication reduction are prioritized.

Despite the significant insights garnered from this study, it is crucial to recognize its limitations. Based on the retrospective design, the study is inherently subject to potential selection bias and uncontrolled confounding factors. Our reliance on historical data may not fully capture all variables influencing the outcomes. For instance, surgical technique, individual surgeon’s experience, and patient adherence to postoperative care instructions, among other factors, were not explicitly considered in this study. These variables can significantly affect the results and thus warrant careful consideration in future studies. Additionally, the study was conducted in a single center, potentially limiting our findings’ generalizability. Different hospitals may have varying standards of care, surgical protocols, and patient demographics, all of which can influence surgical outcomes. Therefore, while our findings provide valuable insights, they should be interpreted cautiously in different healthcare settings. Another significant limitation is the lack of long-term outcome data. While we assessed immediate postoperative outcomes such as operation time, bleeding amount, and postoperative pain, we did not evaluate the long-term effects of these materials on patient recovery and quality of life. For instance, it would be beneficial to know if the reduced postoperative pain in the PRP Group translated into a quicker return to normal activities or better sleep quality over time. Such information would provide a more comprehensive understanding of the impact of these materials in UPPP.

Given these limitations, future research should focus on conducting multicenter, prospective studies with larger sample sizes. Such studies would provide more robust evidence and allow for more generalizable findings. Additionally, future research should incorporate long-term follow-up to assess the lasting effects of these materials on patient recovery, quality of life, and, precisely, sleep apnea symptoms. It would also be interesting to explore the potential biological mechanisms underlying the differential effects of these materials, which could further guide their application in UPPP. Furthermore, cost-effectiveness analyses would be valuable additions to future research. Given the financial implications of healthcare decisions, understanding the cost versus benefit of different functional materials in UPPP could provide further insights into their practicality and affordability. This aspect is particularly pertinent in the current healthcare climate, where cost-effectiveness is critical in clinical decision-making.

This current study reveals the substantial influence of functional material choice on the outcomes of UPPP. The results underline Tissue Glue and PRP’s distinct advantages, emphasizing their potential roles in enhancing surgical outcomes and patient recovery. Our study results also underscore the importance of personalized treatment planning in UPPP. Given the differential impact of various functional materials, surgeons must consider each patient’s specific needs and context when deciding on the material to be used. For example, a patient with a higher risk of bleeding may benefit more from tissue glue, while a patient particularly sensitive to pain might be better served with PRP. This level of personalized care could significantly enhance patient satisfaction and outcomes.

Based on our findings, we recommend that surgeons consider the differential impacts of these materials when planning UPPP procedures. The choice between tissue glue and PRP should be guided by each patient’s specific needs and context, aiming to improve patient outcomes and satisfaction. However, we also emphasize that these findings should be confirmed in future prospective studies before they can be widely adopted into routine clinical practice.

## 5. Conclusions

In summary, this investigation provides valuable perspectives on the significance of biomaterials in surgical results. The selection of materials during UPPP has a significant impact on surgical outcomes and holds great importance in personalized treatment planning. The utilization of Tissue Glue resulted in the shortest operation duration with minimal intraoperative bleeding, indicating its potential advantages for enhancing surgical efficacy and controlling bleeding. It is worth noting that PRP employment exhibited superior outcomes concerning postoperative pain management and complication reduction, which necessitates further exploration to fully comprehend the precise mechanism behind PRP’s effectiveness.

## Figures and Tables

**Table 1:** Patient Demographics.

	None Use	Tissue Glue	PRP	PGA Sheet
**AGE**	38.02 ± 12.31	38.57 ± 11.54	38.64 ± 9.81	39 ± 4.24
**GENDER (M:F)**	83:49	34:12	23:10	2:0
**BMI**	26.68 ± 4.87	27.44 ± 5.82	27.09 ± 4.41	25.87 ± 2.64
**Hemoglobin**	14.50 ± 1.43	14.35 ± 1.53	14.51 ± 1.24	13.35 ± 1.77
**Platelet**	267.54 ± 61.36	269.24 ± 50.80	272.73 ± 60.94	268.00 ± 107.48
**PT**	12.76 ± 0.80	12.95 ± 0.76	12.75 ± 0.71	13.15 ± 0.64
**APTT**	34.17 ± 4.20	32.63 ± 3.72	30.94 ± 2.46	27.50 ± 2.55

## Data Availability

Not applicable.
